# Multilingual text categorization and sentiment analysis: a comparative analysis of the utilization of multilingual approaches for classifying twitter data

**DOI:** 10.1007/s00521-023-08629-3

**Published:** 2023-05-08

**Authors:** George Manias, Argyro Mavrogiorgou, Athanasios Kiourtis, Chrysostomos Symvoulidis, Dimosthenis Kyriazis

**Affiliations:** University of Piraeus, Piraeus, Greece

**Keywords:** Multilingual classifiers, Transfer learning, Zero-shot classification, Transformers

## Abstract

Text categorization and sentiment analysis are two of the most typical natural language processing tasks with various emerging applications implemented and utilized in different domains, such as health care and policy making. At the same time, the tremendous growth in the popularity and usage of social media, such as Twitter, has resulted on an immense increase in user-generated data, as mainly represented by the corresponding texts in users’ posts. However, the analysis of these specific data and the extraction of actionable knowledge and added value out of them is a challenging task due to the domain diversity and the high multilingualism that characterizes these data. The latter highlights the emerging need for the implementation and utilization of domain-agnostic and multilingual solutions. To investigate a portion of these challenges this research work performs a comparative analysis of multilingual approaches for classifying both the sentiment and the text of an examined multilingual corpus. In this context, four multilingual BERT-based classifiers and a zero-shot classification approach are utilized and compared in terms of their accuracy and applicability in the classification of multilingual data. Their comparison has unveiled insightful outcomes and has a twofold interpretation. Multilingual BERT-based classifiers achieve high performances and transfer inference when trained and fine-tuned on multilingual data. While also the zero-shot approach presents a novel technique for creating multilingual solutions in a faster, more efficient, and scalable way. It can easily be fitted to new languages and new tasks while achieving relatively good results across many languages. However, when efficiency and scalability are less important than accuracy, it seems that this model, and zero-shot models in general, can not be compared to fine-tuned and trained multilingual BERT-based classifiers.

## Introduction

With the tremendous growth of the usage and popularity of the social networks in the modern digital societies during the last decade the role of the implementation and utilization of natural language processing (NLP) solutions is more demanded than ever. It is worth noting that in October 2022 the number of people worldwide using social media was 4.74 billion, a 59.3% of the world's population, with 190 million new users joining social media from last year at this time. The latter equates to an annual increase of 4.2% compared to the same period in 2021 [1]. Especially during the COVID-19 pandemic, social media has increasingly assumed a key role in providing information, spreading news and advertising [2] and has become one of the most effective digital marketing tools, with more companies embracing the power of social media analysis [2, 3]. However, on the other hand, it has been highlighted in recent research works that only a small amount of this large portion of data gives added value, making the retrieval of valuable knowledge from this data a challenging task [4, 5].

What is more, another issue that modern social analysis tools face is highlighted by the aspect of multilingualism that characterizes these data. While English is the most used language, however relevant brand, product, or service information can be provided in a variety of languages especially when the examined item is of worldwide interest and consumption. The latter limits the use of supervised machine learning models as well as of monolingual text classification approaches to extract and analyze information due to the lack of high-quality annotated and labeled data and corpora in languages other than English to train these specific models. Thus, in recent years, the implementation of multilingual tools has steadily increased through the utilization of approaches from the domain of artificial intelligence (AI) and more specifically from the domain of deep learning (DL). To achieve multilingual text classification one of the most common approaches is based on the translation of the original text to English. Afterward, a monolingual text classification model, either text categorization or sentiment analysis, is applied on the translated data. However, this approach has revealed a significant loss in the sentiment of the examined data [6, 7]. Another approach is to use sentiment lexicon translated into multiple languages. The main drawbacks of this approach are that it requires the involvement of humans in the process of text analysis, as well as that several lexical objects lack of domain interoperability, thus lexicon-based analysis does not have high accuracy yet and its optimization is still an ongoing research topic [8]. In recent years, the performance of multilingual classification tools has improved and enhanced through the utilization of approaches and techniques that are mainly based on the implementation of transformers [9, 10].

To this end, this paper leverages the potentials that these emerging technologies pose to implement a comparative analysis for achieving multilingual text and sentiment classification on multilingual data. This comparative analysis seeks to highlight the added value and emerging need of the implementation and utilization of multilingual classifiers on multilingual corpuses for two different text classification, the text categorization and the sentiment analysis. In this context different BERT (bidirectional encoder representations from transformers)-based multilingual models were exploited. What is more, a zero-shot classification approach is also implemented in the context of this paper to further evaluate the application of a model that has not been trained before on the data. The overall evaluation and comparison of the first approach is performed through the training of the proposed BERT-based models to open labeled data and reviews related to Amazon products and then applied to raw unlabeled tweets written in different languages, thus achieving zero-shot and transfer inference in terms of model accuracy. While the zero-shot classification approach is utilized without pre-training on both the labeled multilingual data and on the unlabeled multilingual tweets.

Thus, the goal of this paper is to evaluate multilingual text classification approaches with a specific focus on real-world applications. Hence, this paper includes contributions such as:

- A comparison of state-of-the-art language models on multilingual setups, evaluated across five languages and two distinct tasks;
- A set of practical recommendations for finetuning readily available language models for text classification; and
- Analyses and discuss the industry-centric challenges and problems that researchers on social media text mining have to face, such as domain mismatch and labeled data availability

The remainder of this paper is structured as follows. Section 2 describes the related work from the domains of multilingual BERT-based classifiers and zero-shot approach. The overall methodology implementation and the approaches that are followed are being presented in Sect. 3. Afterward, Sect. 4 presents the experimental setup, the datasets that were used and the achieved performances and results of the utilization of the proposed approaches. While in Sect. 5 the overall experimental results are further analyzed and discussed. Finally, Sect. 6 concludes the paper and outlines some directions for future works and further enhancements on the proposed methodology.

## Related work

One of the main challenges facing the modern field of NLP is the need for the implementation of sophisticated, holistic, and multilingual approaches in modern multilingual and multicultural societies. Therefore, researchers are constantly trying to develop the most comprehensive multilingual systems. To this end, several AI research teams from major pioneers, such as Google AI and Facebook AI Research, have introduced multilingual tools, corpora and sentence encoding models that are able to cover any language, thus overcoming the limitations imposed by the lack of labeled data in all languages [11, 12]. Key cornerstones to the overall implementation of multilingual approaches are the utilization of multilingual sentence embeddings and of multilingual classifiers which are further based on pre-trained models and on the approach of transfer learning.

### Multilingual sentence embeddings

Text classification models use word embeddings, or words represented as multidimensional vectors, as their base representations for better capturing the semantic information and for enhancing the language understanding. In this context, several research works have developed multilingual settings by utilizing the English embedding space as a base for the multilingual word embedding space [13, 14]. While these existing multilingual approaches perform well overall in multiple languages, they often underperform in high-resource languages compared to exclusively bilingual models. Furthermore, due to the limited capacity of the model and the poor quality of the training data for low-resource languages, it can be difficult to scale multilingual models to support a larger number of languages while maintaining good performance.

To this end, recent approaches go beyond word embeddings to improve multilingual NLP and capture more semantic meaning by using embeddings of higher-level structures such as sentences or even paragraphs. Existing approaches for generating such embeddings, like LASER [15] or MUSE [16], rely on parallel data, mapping a sentence from one language directly to another language in order to encourage consistency between the sentence embeddings. In addition, recent efforts to improve language models include the development of masked language model (MLM) pre-training [17]. This approach has led to outstanding enhancements across a wide range of languages and a variety of NLP tasks since it only requires monolingual text. In addition, MLM pre-training has been extended to the multilingual setting by modifying MLM training to include concatenated translation pairs, known as translation language modeling (TLM), or by simply introducing pre-training data from multiple languages [18].

In addition, authors in [19] present a multilingual BERT embedding model, called LaBSE, that produces language-agnostic cross-lingual sentence embeddings for 109 languages. The model is trained on 17 billion monolingual sentences and 6 billion bilingual sentence pairs using MLM and TLM pre-training, resulting in a model that is effective even on low-resource languages for which there is no data available during training. Finally, authors in [11] introduce a new evaluation framework for multilingual document classification in eight languages and provide the baselines for all language transfer directions using multilingual word and sentence embeddings.

### Multilingual classification

One of the latest milestones in the NLP field is the introduction of BERT that enables transfer learning with large language models reaching the state-of-the-art for a great number of NLP tasks and applications [10]. In this context several research works have proposed multilingual models based on the utilization of BERT for a wide range of cross-lingual transfer tasks. More specifically, advances in multilingual language models such as multilingual BERT (mBERT) [20] and XLM-RoBERTa [21] which are trained on huge corpus in over 100 languages indicate promising approaches and solutions for the implementation of multilingual applications. In the same context, several research works have introduced multilingual BERT-based models specifically pre-trained on tweet corpora [22, 23]. In theory, cross-lingual approaches reduce the need for labeled training data in target languages by enabling zero- or few-shot learning. Additionally, they enable simplified model deployment compared to the use of many monolingual models. On the other hand, evaluations show that scaling to more languages results in a decrease in the performance of models and thus report the relative underperformance of multilingual models on monolingual tasks [24]. Meanwhile, benchmarks for supervised multilingual text classification are limited. Authors in [15] propose language-agnostic sentence representations (LASER) and evaluate them on multilingual document classification corpus (MLDOC) [11]. However, authors in [25] indicate that their multilingual finetuning and bootstrapping approach, MultiFit, outperforms LASER and mBERT on CLS and MLDOC.

### Multilingual versus monolingual classifiers

The latest indicates that one of the key challenges for the implementation of improved classifications in modern NLP systems is whether to apply and utilize a multilingual classifier in contrary to a monolingual one under the scopes of a research work or task. The emerging utilization of transformers and specifically of BERT led to the deployment of different monolingual language-specific models for many languages based on BERT’s architecture and pre-training procedure. In that context, many monolingual BERT variants have been recently implemented, such as for Chinese [26], Danish [27], Greek [28], and Dutch [29] among others. On top of this, several recent research works attempt to compare the performance between monolingual and multilingual models on specific target languages and downstream tasks. The outcomes indicated that certain monolingual models may outperform as compared to multilingual ones as they can leverage more specialized and extensive language models that are tailored to the specific target language [30, 31]. The latter is further supported and enhanced specifically in cases that monolingual tokenizers have been trained by native-speaking experts who are aware of relevant linguistic phenomena applying on their target language [32]. Thus, the monolingual models also provide better sentence representations. However, these sentence representations are usually domain-specific and do not generalize well across different tasks and especially on tasks that are based on the utilization of multilingual datasets, where also the utilization of several different monolingual models would be proven an extremely time-consuming approach. The latter highlights the need for better and more generalized sentence embedding models and is highly addressed by the utilization of multilingual models [33]. At the same time multilingual approaches have proven to be more accurate in scenarios that are based on multilingual datasets and that target their outcomes on multiple languages rather than on a specific one [34]. In such cases, using a multilingual model can enable the model to handle the nuances and variations across different languages, and make more accurate predictions leading to better performance. By training a model on multiple languages, it can learn from the similarities and differences between languages and improve its overall performance on all languages leveraging its transfer learning capabilities [35]. This can be especially useful for low-resource languages, where it may be difficult to train a high-quality monolingual model due to the lack of training data. In that direction, a research work has indicated that monolingual BERT underperforms mBERT on low-resource languages and suggests that mBERT multilingual training benefits low-resource languages by transferring knowledge from its training on other languages [36]. Another key outcome of this research work is that pairing linguistically related languages can benefit representation learning and adding extra languages can further improve the performance of a mBERT model. To conclude, monolingual models are not superior to multilingual ones per se, but they gain advantage in direct comparisons by incorporating more pre-training data and using language-adapted tokenizers. Complementary to this, especially on text classification tasks that leverage multilingual datasets, such in the case of texts derived from social media platform as in this research work, using a multilingual model it will decrease the execution time, as well it may provide better accuracy and performance than using a monolingual one.

### Multilingual classification on tweets

Recently multilingual classifiers that base their implementation on BERT pre-trained models and architecture have proven to perform very well on NLP downstream tasks related to Twitter messages. These tasks range from text categorization [37] to sentiment analysis [38], and from topic modeling [39] to named entity recognition [40]. Training multilingual classifiers to identify sentiment, topics, named entities, and other features across multiple languages in tweets enables researchers and businesses to gain a more comprehensive and cross-border understanding of what people are globally discussing and what their sentiments are about different topics, products, and services. Moreover, several multilingual BERT-based models have been especially pre-trained on tweets to further enhance their functionality and wide applicability on them. In that context, the XLM-T models was introduced that is a large multilingual Twitter-specific language model based on XLMR checkpoints and architecture specifically pre-trained on millions of tweets in over thirty languages [22]. In addition, the Twitter Heterogeneous Information Network (TwHIN) was introduced recently [41]. A model that has been trained on 7 billion tweets in over 100 different languages and has been fine-tuned on a variety of multilingual social recommendation and semantic understanding tasks. Its utilization and evaluation on a series of different tasks has showcased that this model outperforms as compared to other BERT-based multilingual models and more specifically to mBERT, XLM-R and XLM-T. In the same direction, authors in [42] introduced and evaluated the Benice model that is a multilingual RoBERTa language model trained from scratch on 2.5 billion tweets consistently and was showcased that it outperforms or matches its closest competitors XLM-T and TwHIN-BERT. The latest achievements indicate that multilingual language models when pre-trained on large and domain-specific datasets tend to outperform the generic multilingual pre-trained models. However, on the other hand they may lack of genericity and wider applicability on other domains and different kinds of datasets, hence more research work should be applied to evaluate their utilization on divergent sectors and data.

### Zero-Shot classification

The utilization of transfer learning techniques leverages the development of multilingual models [43]. On top of this, recent research works have been applied on a specific form of transfer learning, the zero-shot learning [44]. The latter is utilized when no training data are available for a model that is used for prediction and with the ultimate objective to minimize the overall latency while maintaining robust classification performances. The main idea is to learn a mapping from classes to a vector in such a way that an unseen class in the future can be mapped to the same space and specific information can be retrieved for this unseen class. To this end, several research works take advantage of this approach and apply to several NLP tasks achieving great results [45, 46].

### Advancements beyond the related work

In this paper a comparative analysis of two different approaches is implemented, to indicate the maturity and accuracy of these approaches in two different tasks of multilingual text classification, the multilingual text categorization and the multilingual sentiment analysis. At first, the approach of BERT-based multilingual classification (BERT-MC) is analyzed and presented as a tool for retrieving semantically similar text in multiple languages. This approach has a twofold interpretation, as it also utilizes and evaluates the use of multilingual sentence embeddings coupled with BERT-based models. Thanks to the ability of multilingual sentence embeddings to represent meanings of words from different languages in the same vector space, the proposed multilingual BERT-MC models become almost independent from the input language. Moreover, exploiting the vector alignment of multilingual sentence embeddings, a language-agnostic pipeline is defined that can be trained with any initial labeled data set, and then applied to Tweets written in different languages. Finally, a zero-shot classification approach is followed, and its outcomes are also evaluated and compared with those of the different implemented and fine-tuned BERT-based models of the BERT-MC approach, thus a comprehensive analysis and comparison between these two approaches is introduced, in both the different multilingual text classification tasks, thus the multilingual text categorization and the multilingual sentiment analysis.

## Methodology and models

**Fig. 1:**
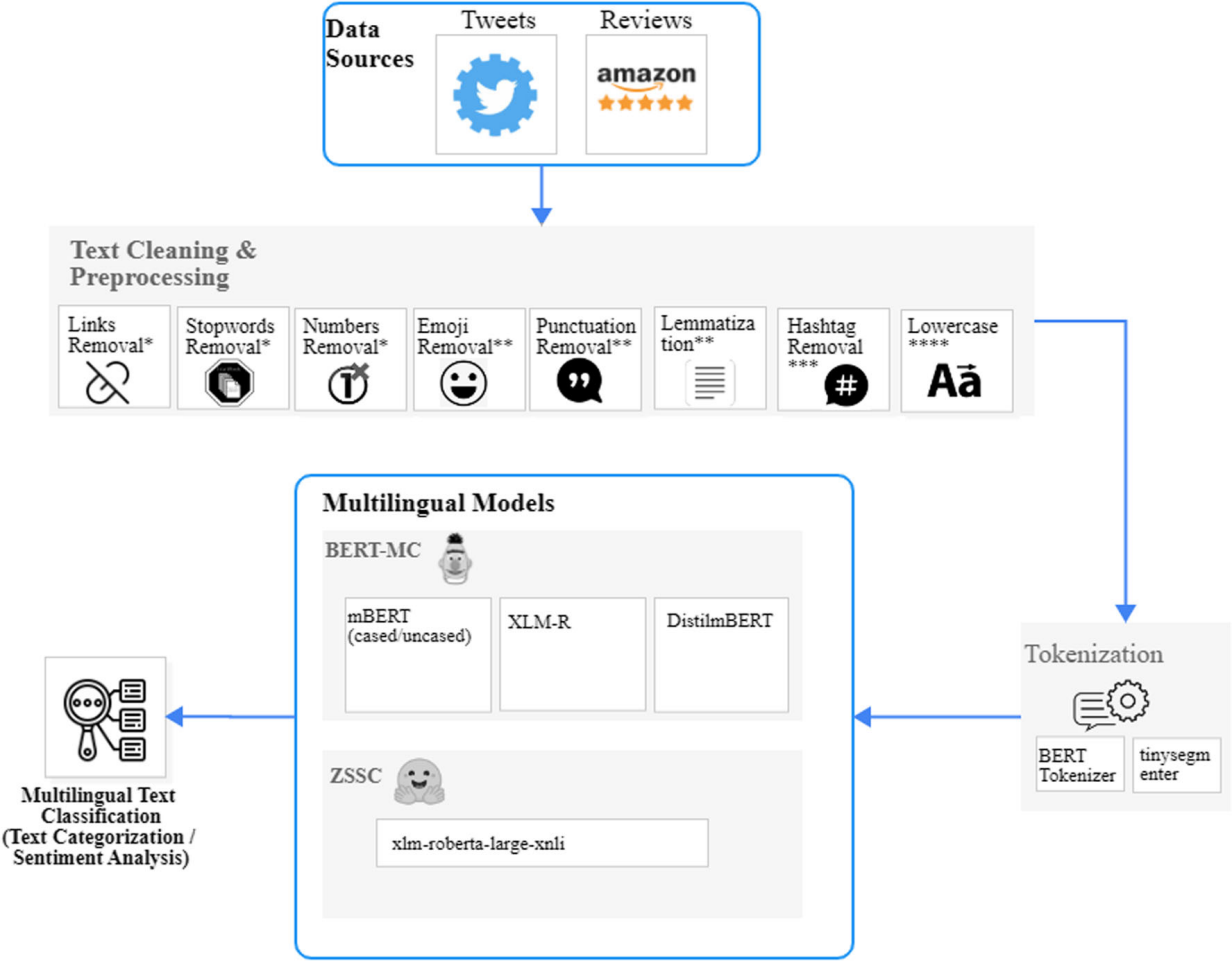
Introduced pipeline for multilingual text classification (* applicable in all scenarios ** applicable only on multilingual text categorization scenarios *** applicable only on multilingual sentiment analysis on Twitter data **** applicable only on the scenario where mBERT uncased is utilized)

Transformer-based models are currently the best performance models in NLP or computational linguistics. The architecture of the transformer model consists of two parts: encoder and decoder. It differs from previous deep learning architectures since it pushes in all the input data at once rather than progressively, thanks to the self-attention mechanism [47]. Following the introduction of BERT from authors in [10], models based on these architectures are emerging and the implementation of several applications is based on them. Challenging NLP tasks have been effectively solved using these models. BERT uses bidirectional encoder representations instead of traditional techniques that only learn from left to right or right to left, the same architecture as a bidirectional recurrent neural network (RNN). A bidirectional architecture includes two networks, one in which the input is handled from start to end and another one from end to start. The outputs of two networks are then integrated to provide a single representation. As a result, BERT is able to better understand the relationship between words and provide better performances. Besides, unlike other context-free word embedding models like word2vec [48] or GloVe [49], which create a single word embedding representation for each word in its vocabulary, BERT needs to consider each context for a given word in a phrase. As a consequence, homonyms in other sentences become different in each context. Language models are usually specifically designed and trained in English, most globally used language. Moreover, these models were then deeper trained and became multilingual to expand and serve NLP problems to support more languages globally. Hence, in this study, various BERT-based models are applied to the examined datasets for solving social media text classification tasks. The overall workflow that has been followed in the context of this study is depicted in Fig. 1, where are presented the preprocessing steps including the text cleaning and tokenization, as well the two different multilingual approaches that are utilized in the context of this research work.

### BERT-based multilingual classification (BERT-MC)

Under the scopes of this approach four different multilingual and domain-generic BERT-based models were utilized and more specifically, two different versions of the mBERT model (cased and uncased), a XLM-R model, and a DistilmBERT model.

*Multilingual BERT (mBERT)* The multilingual BERT (mBERT) is an updated version of BERT that is trained on 104 different languages as introduced in [10], including uncased and cased versions, after the launch of BERT. The multilingualism that characterizes this model facilitates the semantic understanding of the relationships between words in all these 104 different languages. Moreover, both the cased and uncased versions of mBERT are utilized in the proposed approach, as the cased version of mBERT is helpful in languages that the accent plays an important role, such as in German language. The mBERT models, as opposed to their predecessors, are trained in various languages and use masked language modeling (MLM). To utilize both the cased and uncased version of mBERT corresponding models from the “*transformers*” library were loaded, and further fine-tuned and utilized as detailed in Sect. 4.4. More specifically, for the mBERT cased the “*bert-base-multilingual-cased*” model was loaded, while for the mBERT uncased version the “*bert-base-multilingual-uncased*” model was loaded.

*XLM-RoBERTa (XLM-R)* XLM-RoBERTa (XLM-R) is a multilingual masked language model introduced by authors in [21] that trained on 2.5 TB of newly created clean CommonCrawl data in 100 languages. It is an update to the original XLM-100 model that was also introduced by the same team [50] and has proven to offer significant gains in downstream NLP tasks such as text classification, and question answering over previously released multilingual models like mBERT or XLM. In the context of this research work the 24-layer XLM-RoBERTa model (“*xlm-roberta-base*”) from the “*transformers*” library was utilized and further fine-tuned in the datasets and tasks presented in this research work.

*DistilmBERT base multilingual* DistilmBERT base multilingual [51] is a distilled version of the mBERT model and is trained on the concatenation of Wikipedia in 104 different languages. The model has six layers, 768 dimension and 12 heads, totalizing 134 M parameters, as compared to the 177 M parameters for mBERT-base model. On average, this model, referred to as DistilmBERT, has proven to be twice as fast as mBERT-base and is considered as a pre-trained smaller general-purpose language representation model. The multilingual DistilmBERT model that was evaluated and further compared with the two previously presented models was based on the “*distilbert-base-multilingual-cased*” model from the “*transformers*” library.

### Zero-shot sentiment classification (ZSSC)

**Fig. 2:**
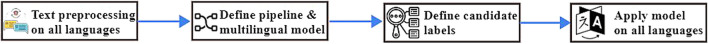
Zero-Shot Sentiment Classification (ZSSC) pipeline

Recently, zero-shot text classification attracted a huge interest as it is an approach that facilitates the prediction of the target class of a text without having trained on any of the candidate classes/labels [52]. To this end, zero-shot classification is a revolution for unsupervised text classification as it provides more flexibility and demonstrates all the power of transfer learning for more general and unlabeled data. In this context, a zero-shot classification pipeline from Hugging Face library was utilized [53]. More specifically, the pipeline module, presented also in Fig. 2, was utilized and the xlm-roberta-large-xnli model was selected as the appropriate one to be utilized and evaluated [54], as it is fine-tuned on 100 different languages on the multilingual XNLI dataset [55]. In contrary to the XLM-R model that was implemented and utilized in the BERT-based approach, this model was applied on following a zero-shot and without a previous pre-training and fine-tuning on the analyzed data. Moreover, as the problem in this research work is a multi-class classification, the “multi_label” parameter was placed on True, thus the scores are independent for each class, but for each of them its probability is computed between 0 and 1.

## Experimental setup

This section presents the experimental results of the utilization of the examined models on the tasks of the multilingual text categorization and the multilingual sentiment analysis both in the labeled multilingual datasets, as well in the unlabeled Twitter data. As already introduced the proposed BERT-MC models are first trained and evaluated on the multilingual amazon reviews corpus (MARC) dataset [56]. This dataset represents the first set of experiments were both abovementioned sub-tasks are implemented. Afterward, in the second set of experiments the already trained BERT-MC are further utilized and evaluated on multilingual Twitter data to categorize them as per their content, thus performing the multilingual text categorization sub-task, and to further score them as per their expressed sentiment, thus performing the multilingual sentiment analysis sub-task. These Twitter data have been collected under the scopes of an evaluation of the proposed models in a real-world scenario to better analyze and understand the key factors influencing consumers purchasing decision on different wine brands and products. With regard to the ZSSC pipeline it is utilized without pre-training in both sets of experiments to examine and evaluate its performance and applicability on data and scenarios in which it has not trained before.

### Datasets

Under the scopes of this research work two different datasets were used as the core objects in which the models are utilized and evaluated. At first, the MARC dataset was used to train and evaluate the models on a widely used and tested large multilingual corpus and evaluate their accuracy and cross-lingual transfer inference.

**Fig. 3:**
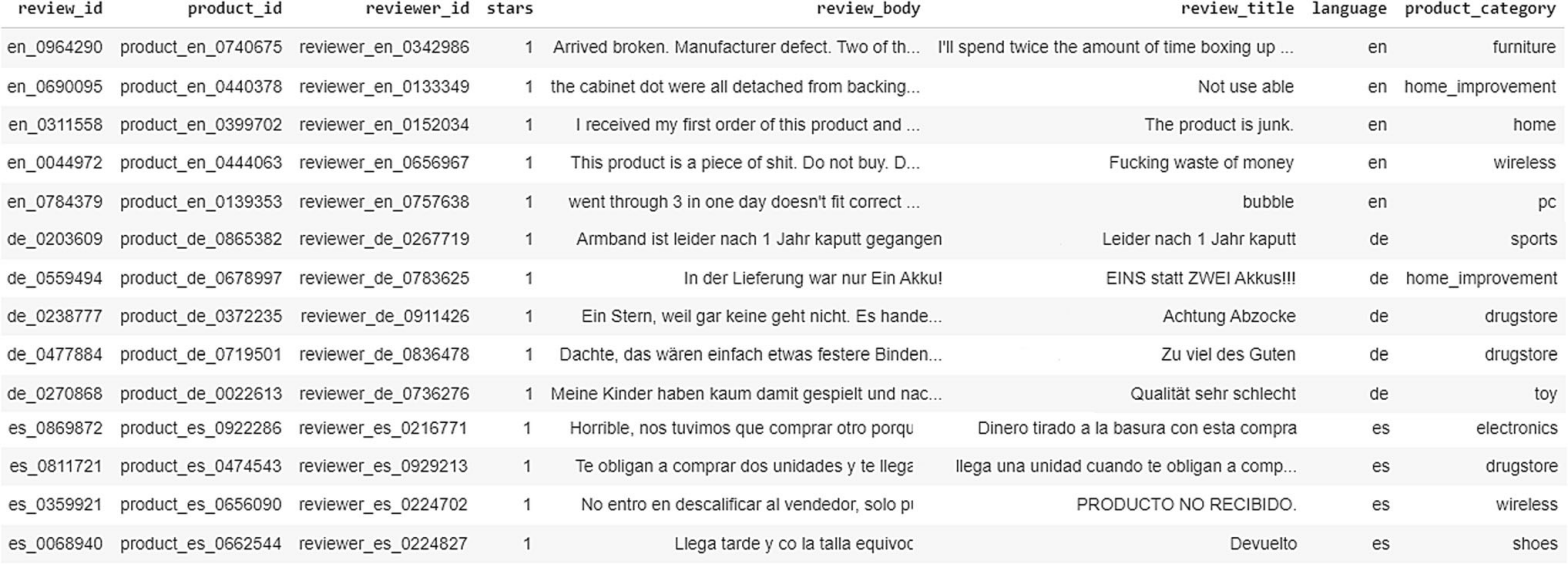
A snapshot of the MARC multilingual data

The initial dataset on which the introduced models were trained and evaluated is a collection of Amazon reviews specifically designed to aid research in multilingual text classification tasks [56]. The dataset contains reviews in English, Japanese, German, French, Chinese and Spanish, collected between November 1, 2015 and November 1, 2019. Each one the records represented into the different language datasets contains an anonymized “review ID”, an anonymized “product ID”, an anonymized “reviewer ID”, the review text as “review_body”, the “review_title”, the star rating as “stars” attribute, the “language” on which the review is written, and the coarse-grained “product_category”, e.g., “furniture”, “sports,” etc. A respective snapshot with corresponding data from 3 languages, i.e., English, German, and Spanish, is depicted in Fig. 3.

**Fig. 4:**
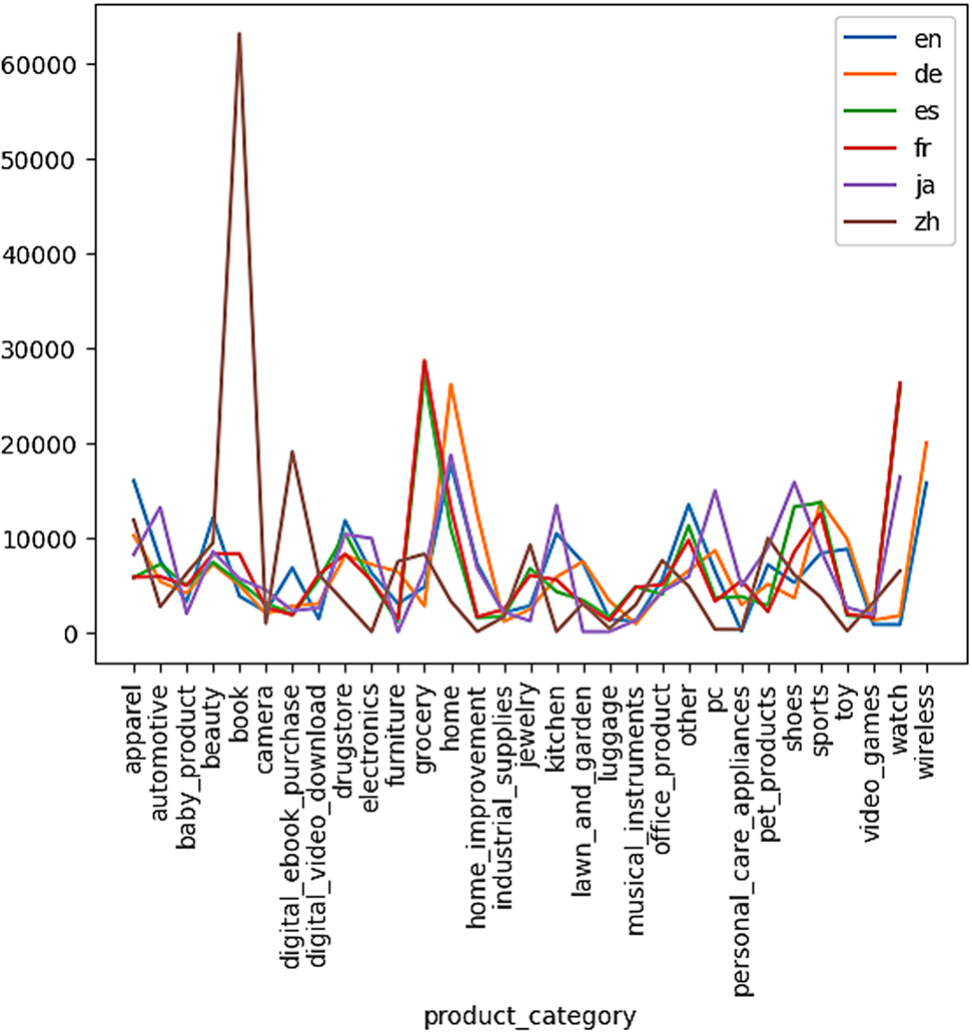
Occurrence of the 31 product categories across the different language datasets of the MARC dataset

Moreover, the corpus is balanced across a 5-star rating system (1 “highly negative” − 5 “highly positive”), so each star rating constitutes 20% of the reviews in each language. For each of the five examined language, there are 200,000, 5000 and 5000 reviews in the training, development, and test sets, respectively. The maximum number of reviews per reviewer is 20 and the maximum number of reviews per product is 20. All reviews are truncated after 2000 characters, and all reviews are at least 20 characters long. Moreover, it should be noted that the number of different product categories represented into the different language datasets are 31 with uneven distributions and occurrences in each of these datasets, as also depicted in Fig. 4. The proposed models were trained and applied on this multilingual dataset to test their accuracy on the identified class, with regard to the multilingual text categorization task, as well on the review sentiment score based on Amazon’s 5-star rating system, with regard to the multilingual sentiment analysis task.

**Fig. 5:**
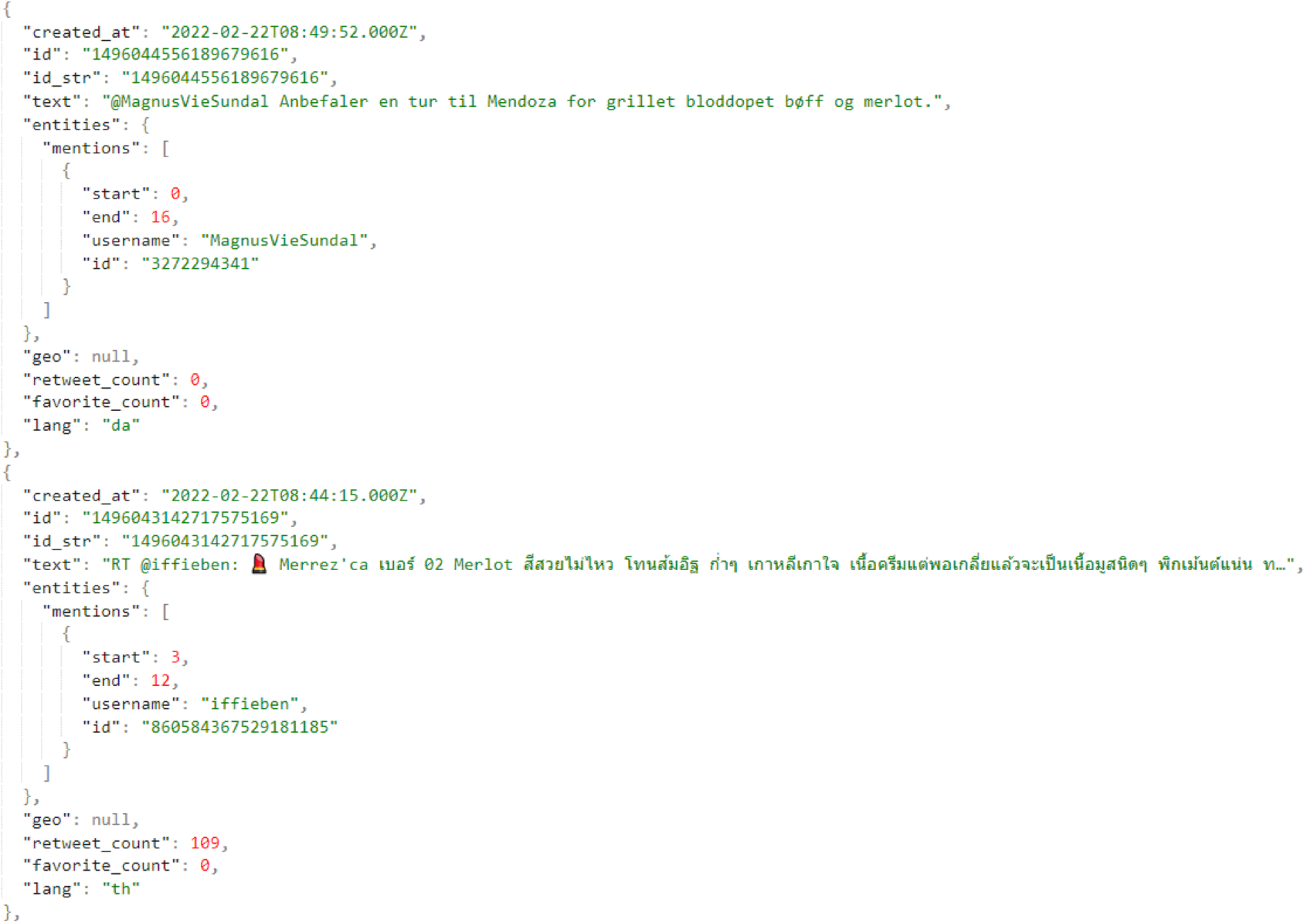
A snapshot of two raw tweets on different languages

Then, a dataset of 11,750 raw and unlabeled tweets was used in order to utilize the examined models and test their transfer learning inference, especially of the trained ones, the BERT-MC. A snapshot of a sample of two respective tweets is depicted in Fig. 5. This dataset was also utilized to evaluate the performance of the models on both the multilingual text categorization task, as well on the multilingual sentiment analysis task. It is of emerging need for a model to accurately classify a raw tweet based on its category/topic, as raw tweets that have been collected based on a specific keyword may not be referred to the same topic. For instance, a batch of tweets that has been collected based on the wine product “bordeaux” as keyword, may belong on several topics and categories, such as to politics, as it refers to a region, to sports, as it refers to a local football team, or even to a wine product, which is the topic of interest in the context of this study. As concerns the task of classifying raw tweets based on the expressed sentiment it is one of most widely researched and of high interest aspects with several research works and widely used implemented tools.

### Evaluation setup and metrics

Experiments presented in this paper were based on implementing the multilingual text classification tasks and the presented approaches with the Python programming language and the utilization of TensorFlow 2.0 [57] and Keras libraries [58]. Moreover, the implementation of the code, for both BERT-MC and ZSSC sets of experiments, was performed on Google Colab [59] online platform to facilitate the overall running time through the usage of the GPU option that is available.

What is more, the metrics considered in the evaluation of the models are accuracy, precision, recall, and F1-score and are defined as below:

- *Accuracy* Measures the sum of the correct predictions, thus true positive (TP) and true negative (TN), made by a model in relation to the total number of predictions made.
  $$a= \frac{TP+TN}{TP+TN+FP+FN}$$
- *Precision* Measures how many of the positive predictions made are correct, thus it represents the ratio of true positive (TP) to the sum of true positive (TP) and false positive (FP).
  $$p= \frac{TP}{TP+FP}$$
- *Recall* Measures of how many of the positive cases the classifier correctly predicted over all the positive cases in the data, thus it represents the ratio of true positive (TP) to the sum of true positive (TP) and false negative (FN).
  $$r= \frac{TP}{TP+FN}$$
- *F1-Score* The F1-score or F1-measure is one of the most widely used measures in NLP and machine learning (ML) tasks, because it provides a way to combine both precision and recall into a single measure that captures both properties. The system is defined as the weighted harmonic mean of its precision and recall, that is:
  $$F= \frac{1}{a*1/p + (1-a)*1/r}$$

F1-score has proven to be a better measure to be used and examined, as it provides a balance between precision and recall.

### Preprocessing

#### Text cleaning

**Fig. 6:**
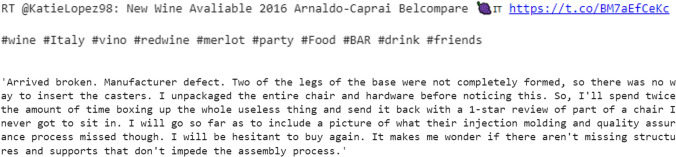
Sample of raw texts from the Twitter and MARC dataset

**Fig. 7:**
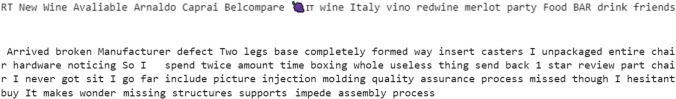
Sample of texts after applying the text cleaning process

Two different preprocessing steps are applied in both the examined multilingual datasets and experimentation sets as also depicted in Fig. 1. The tokenization of the examined data and the text cleaning. At first, all raw data in both the MARC and Twitter datasets are cleaned. In that context, different text cleaning strategies are implemented based on the different tasks as well as on the different characteristics of the datasets. As each used dataset is distinct in terms of vocabulary, origin, and content, thus different preprocessing approaches are appropriate for each dataset before feeding data into the models, as summarized in Table 1. More specifically, numbers, URLs and stopwords were removed in all sub-tasks, while for the multilingual sentiment analysis sub-task punctuations, emojis, and emoticons are not removed because, in several cases, users often use them in their comments to express their sentiment. On the other hand, as concerns the multilingual text categorization sub-task in both the experimentation sets further lemmatization and punctuations and emojis removal was applied on the raw reviews and tweets. Representative snapshots of the data before and after the utilization of the text cleaning are depicted for both the examined datasets in Figs. 6 and 7, respectively. Finally, the texts have been lowercased only before the application of the mBERT uncased version. To successfully perform all these actions, the proposed pipeline exploits the library of *NLTK* [60]. More specifically, NLTK library and its corresponding components were deployed for applying stopwords and punctuation removal, as well for lowercasing the cleaned text in the cases that each one of these actions was applicable as indicated in Fig. 1.

**Table 1:** Preprocessing techniques applied in each experiment

	Links	Stopwords	Number	Emoji	Punctuation	Lemma	TweetsHashtag	Lowercase	Tokenization
	*Multilingual text categorization*								
mBERT (c)	V	v	v	v	v	v	x	x	v
mBERT (un)	V	v	v	v	v	v	x	v	v
XLM-R	V	v	v	v	v	v	x	x	v
DistilmBERT	V	v	v	v	v	v	x	x	v
	*Multilingual sentiment analysis*								
mBERT (c)	V	v	v	x	x	x	v	x	v
mBERT (un)	V	v	v	x	x	x	v	v	v
XLM-R	V	v	v	x	x	x	v	x	v
DistilmBERT	V	v	v	x	x	x	v	x	v

#### Tokenization

Afterward, before the examined and cleaned texts can be fed to the proposed models, a specific preprocess step should be applied on them, the tokenization. This step is accomplished by using off-the-shelf BERT-based tokenizers for each model, respectively, and more specifically the BERT Tokenizer, the XLM-RoBERTa tokenizer and the DistilmBERT tokenizer. The reason of using different tokenizers is that the XLM-R and DistilBert architectures differs from the mBERT, as they do not make use of token type ids, thus the BERT tokenizer which will tokenize the inputs by creating meta-features and by converting the tokens to their corresponding IDs is not applicable these two models. To support the functionality of the tokenization the *AutoTokenizer* class from the *“transformers”* library was utilized and the “*bert-base-multilingual-cased*”, “*bert-base-multilingual-uncased*”, “*xlm-roberta-base*” and “*distilbert-base-multilingual-cased*” were utilized, respectively, in the four examined moder, i.e., mBERT (cased), mBERT (uncased), XLM-R and DistilmBERT. The only exception is applied on the Japanese data of the MARC dataset where the tinysegmenter^1^ is utilized as also proposed by authors in [61].

### Models training and utilization

As stated in Sect. 3.1 the evaluation of all BERT-based multilingual models was based on the utilization of the “*transformers*” library. As a consequence, corresponding classes from these two libraries were utilized for further train and finetune the models before their final utilization and evaluation. It should be noted that the training of the models was performed in the MARC dataset and that the “*TrainingArguments*” and “*Trainer*” classes were used from the “*transformers*” library. At first, the pre-trained models were loaded, and dedicated instances created for each model. The number of labels was set accordingly to the candidate labels for each task, thus to 31 with regard to the Multilingual Text Classification task as the number of different categories indicated in the MARC dataset, and to 5 with regard to the Multilingual Sentiment Analysis task based on Amazon’s 5-star rating system. Then the same training hyperparameters were initialized for all the models based on the “*TrainingArguments*” class and more specifically, “*evaluation_strategy*”: “*epoch*”, “*num_train_epochs*”: *5*, “*learning_rate*”: *2e-5*, and “*optim*”: “*adamw_hf*” to utilize the Adam optimizer.

The fine-tuning of the models was finalized by utilizing the “*Trainer*” class, setting the examined model, the training hypermeters that were set before and by using the 20% of labeled reviews of the MARC dataset as the training set of the employed dataset. The resulted trained model was utilized in the next set of experiments for each specific task.

## Analysis and discussion

As analyzed in previous sections two different sets of experiments are implemented to further evaluate the proposed approaches and models. At first, the MARC dataset was used that contains 200,000, 5000 and 5000 reviews in the training, development, and test sets, respectively, for each one of the six languages (English, German, French, Spanish, Japanese and Chinese) that are included in these corpora. The training sets were used as the training datasets for the evaluation of the proposed approaches, while the concatenation of the development, and test sets was used for the evaluation of the accuracy of the introduced models. It should be noted that the models were trained and evaluated in two different iterations, representing the two different sub-tasks on which they are utilized and evaluated, the multilingual text categorization and the multilingual sentiment analysis. The results in Table 2 indicate that the three models of the BERT-MC approach can achieve good performances when trained and utilized on the multilabel text categorization task, as also on the multilingual sentiment analysis. More specifically, the XLM-R model outperforms all the other models in both the examined iterations and sub-tasks. Of course, further fine-tuning and different settings in the selected layers and the activation functions may enhance their performances. On the other hand, the ZSSC approach fails to generalize well and performs pure in both the examined iterations and sub-tasks as it fails to capture the semantics for various candidate labels, as in these experiments. More specifically, as concerns the multilingual text categorization sub-task, 31 different labels exist, while as concerns the multilingual sentiment analysis sub-task, 5 different candidate labels exist based also on the Amazon’s Star Rating System.

**Table 2:** Experimental results for the MARC dataset

Model	Precision	Accuracy	Recall	F1-score
*Multilingual text categorization*				
mBERT (cased)	0.6820	0.6813	0.6993	0.7004
mBERT (uncased)	0.6954	0.7002	0.6989	0.7075
XLM-R	0.7303	0.7379	0.7332	**0.7342**
DistilmBERT	0.6861	0.6782	0.6834	0.6792
ZSSC	0.4705	0.4686	0.4741	0.4703
*Multilingual sentiment analysis*				
mBERT (cased)	0.7201	0.7286	0.7301	0.7183
mBERT (uncased)	0.7303	0.7372	0.7341	0.7354
XLM-R	0.7662	0.7585	0.7603	**0.7642**
DistilmBERT	0.7161	0.7182	0.7234	0.7192
ZSSC	0.5156	0.5231	0.5156	0.5214

Finally, the already implemented models are evaluated on real-world multilingual Twitter data that have been fetched based on specific keywords related with different kinds of wine products and more specifically the keywords “*merlot*”, “*cabernet*”, “*bordeaux*”, “*chardonnay*”, “*malbec*” and “*carinena*”. The outcomes of the application of the examined models in multilingual tweets have shown remarkable results and have indicated that the implemented BERT-models generalize very well. Table 3 depicts five sample tweets from the overall 11,750 collected ones that represent a good performance and classification from the application of these models on both the examined iterations and different sub-tasks. In this experiment, it also worth mentioning that the ZSSC approach gives close results and classifies the examined tweets in close or same classes as the BERT-MC approach, as the candidate labels are relatively low than in the first set of experiments especially as concerns the Multilingual Text Categorization sub-task, where only five candidate labels were set (“politics”, “business”, “sports”, “world”, and “wine”) as compared to the 31 from the MARC dataset. The latter indicates the need for specific fine-tuning of the initial ZSSC model to further introduce weights in the candidate labels. Finally, it should be noted that as the analyzed raw tweets are unlabeled and in order to better evaluate the outcomes of the examined models from the respective stakeholders, a manual-human labeling was performed on the tweets by relevant stakeholders and policy makers as this application refers to a real-world scenario as introduced in the context of the PolicyCLOUD project [62]. The objective of this scenario is to provide both policy makers and private actors/stakeholders with effective data-driven analysis and insights to understand consumer demands and identifying trends in the world of wine. More specifically, the manual-human categorization was performed only on 3500 tweets and the manual-human sentiment scoring was performed only on 500 tweets that were categorized by the human annotators/stakeholders as related to the “wine” category. The latter facilitates in the evaluation of the examined tweets even from a manual and human way to enhance the policy making procedures and the extraction of actionable knowledge.

**Table 3:** Sample classifications for the multilingual tweets

Original tweet	Language	Model	Predicted class
*Multilingual text categorization*			
RT @clembsz: bon jfais ma pub ici aussi, si t’es de bordeaux, que t’as envie de ressembler à un cahier de brouillon, et que mon style te pl	French	mBERT (cased)	World
		mBERT (uncased)	World
		XLM-R	World
		DistilmBERT	Wine
		ZSSC	Wine
		Manual-Human	Business
RT @wine_is_art: Three Left Bank of #Bordeaux facts\n\n• It’s about powerful long-lived wines.\n• Cabernet Sauvignon is the main grape variety	English	mBERT (cased)	Wine
		mBERT (uncased)	Wine
		XLM-R	Wine
		DistilmBERT	Wine
		ZSSC	Wine
		Manual-Human	Wine
Le RDV emploi/formation à ne pas manquer ! 150 employeurs seront présents le 24 novembre pour la 4 ème édition du Carrefour Emploi Bordeaux Métropole au Hangar 14 et en ligne jusqu’au 28 novembre !	French	mBERT (cased)	Business
		mBERT (uncased)	Business
		XLM-R	Business
		DistilmBERT	Business
		ZSSC	Business
		Manual-Human	Business
Todas las crónicas de Tercera RFEF: El Binéfar castiga al Cariñena por sus minutos de desconexión	Spanish	mBERT (cased)	Politics
		mBERT (uncased)	Politics
		XLM-R	World
		DistilmBERT	World
		ZSSC	World
		Manual-Human	World
Locura en Cariñena con un centenario Binéfar desencadenado tras el descanso. Partido loco, 4 goles, 3 penaltis, 1 larguero… del 2–1 al 3–4 en menos de 15 minutos	Spanish	mBERT (cased)	Sports
		mBERT (uncased)	Sports
		XLM-R	Sports
		DistilmBERT	Sports
		ZSSC	Sports
		Manual-Human	Sports
*Multilingual sentiment analysis*			
@MagnusVieSundal Anbefaler en tur til Mendoza for grillet blondet bøff og merlot	Danish	mBERT (cased)	4
		mBERT (uncased)	4
		XLM-R	4
		DistilmBERT	4
		ZSSC	3
		Manual-Human	4
RT @wine_is_art: Three Left Bank of #Bordeaux facts\n\n• It’s about powerful long-lived wines.\n• Cabernet Sauvignon is the main grape variety	English	mBERT (cased)	5
		mBERT (uncased)	5
		XLM-R	5
		DistilmBERT	4
		ZSSC	4
		Manual-Human	5
RT @KatieLopez98: New Wine Avaliable 2016 Arnaldo-Caprai Belcompare t.co/BM7aEfCeKc\n\n#wine#Italy#vino#redwine#merlot#party#Food#BAR#drink#friends	English	mBERT (cased)	2
		mBERT (uncased)	2
		XLM-R	3
		DistilmBERT	2
		ZSSC	1
		Manual-Human	3
@PaulEindbaas Is dat een flesje Merlot ernaast voor de nodige inspiratie?	Dutch	mBERT (cased)	3
		mBERT (uncased)	3
		XLM-R	3
		DistilmBERT	2
		ZSSC	1
		Manual-Human	3
Para compensar un mal lunes, un buen #vino de @altavins @doterraalta\n\nHoy Tempus 2016. Garnacha, Syrah, Merlot y Cariñena. 12 meses en barrica. \n.\n\n#mivinodehoy	Spanish	mBERT (cased)	5
		mBERT (uncased)	5
		XLM-R	5
		DistilmBERT	5
		ZSSC	4
		Manual-Human	5

## Conclusion and future work

Text classification is an important task of NLP on the procedures of modern businesses and organizations as it facilitates the extraction and acquisition of new insights and knowledge from data and automates business processes. While the implementation and utilization of efficient and accurate multilingual NLP solutions is of high importance these days due to the high multilingualism that describes our modern digital societies. To address a portion of these challenges, this paper seeks to highlight these two aspects and emerging needs by conducting a comparative analysis and evaluation of different multilingual approaches on two different experimentation sets. The latter is implemented with specific emphasis on the utilization and evaluation of the examined multilingual models on two different sub-tasks of the text classification task, the multilingual text categorization and the multilingual sentiment analysis sub-tasks that are both of high research and business interest during the last years. Moreover, the implemented models are utilized and evaluated in terms of their accuracy on two different multilingual datasets, the MARC dataset [56] and a multilingual dataset including data that were collected from the Twitter based on identified keywords highly related to a real-world scenario as it has been introduced in the context of the PolicyCLOUD project [62]. Through this approach this study achieves the introduction of an extensive and novel comparative analysis as the models are utilized and evaluated also in a real-world scenario and on a dataset that has not been examined before by the research community and demonstrates the applicability of the examined models on such a scenario. The MARC dataset was first selected to train and evaluate the performance of the examined models as it provides limitation in the words and characters that appear in a review. More specifically, all reviews are truncated after 2000 characters. A characteristic that makes the dataset alike to the Twitter dataset, as compared to other datasets that include long texts, due to the limitation on characters that expresses tweets. Hence, the models are trained on texts and sentences with few words and are considered appropriate candidates to be then utilized and evaluated in Twitter data. On top of this, one of the main contributions of this research work is the examination of the role and the impact of the pre-trained multilingual sentence embeddings on the two above-mentioned multilingual sub-tasks. Through this approach the fundamental characteristics of transfer learning are analyzed, as the utilization of multilingual sentence embeddings along with pre-trained BERT-models can facilitate the implementation and application of the proposed models in different languages than in the initial language in which they were trained on with almost zero losses. The latter also indicates that models learned and trained on labeled data with respective information provided, as in the experimentation set of the MARC dataset, can perform better than models trained in unsupervised conditions. Hence, the transfer of the knowledge from one language to another set of languages, or even from another domain to another, can be accomplished without significant losses.

At this point, it should be noted that as concerns the multilingual text categorization sub-task a different number of product categories was used as candidate labels in the utilization of the models on the real-world Twitter dataset as compared to the MARC dataset. More specifically, during the second set of experiments that based on the Twitter dataset only five categories were used as candidate labels in comparison to the initial 31 categories that are candidate labels in the MARC dataset. On the other hand, with regard to the multilingual sentiment analysis sub-task the Amazon’s 5star rating system was used in both the different experimentation sets and datasets. Furthermore, the ZCCH model was not initially trained, rather than a total zero-shot classification was chosen to be performed to indicate the maturity of this tool and pipeline to be applied without pre-training and to compare its accuracy with the already trained BERT-MC models. To this end, further fine-tuning, and pre-training of this ZCCH model may lead to enhanced performance, better accuracy and results similar to them of the BERT-MC models. Moreover, during the second experimentation set the manual-human categorizations and especially the manual-human sentiment scorings were not initially performed and were applied on the processed tweets only after the utilization of the models and in a specific number of Tweets. Thus, the implementation of a manual annotation and labeling task as one the first steps of the processing of the Tweets can be used as an appropriate method for the enhancement of the models, as the models can be trained and fine-tuned specifically on these data and information. This approach can be useful especially in the cases where the texts need to be classified in several different classes/labels as in the Multilingual Text Categorization sub-task. In that context several annotation methodologies will be researched and applied in the future [63], as human annotators have indicated as a good approach especially on relatively small datasets as in second experimentation set of this study [64].

What is more, this research work focuses on the analysis of data highly related with products and with regard to the Twitter data specifically focused on the agricultural and food technology domains, as the data have been collected based on different kinds of wines, such as “merlot” and “chardonnay”, as keywords and not on a wider domain and area of interest, as telecommunications or smart cities. The wider applicability of the examined models and their evaluation on broader domains is also an aspect of future work. The latter will provide actionable knowledge to interested stakeholders to further enhance their policies based on the multilingual sentiment analysis task on specific wine products. In the context of this research work a comparative analysis between domain-generic multilingual pre-trained models has been performed to research and evaluate their scalability, generalization, and performance on datasets from a domain, like this of the social media and specifically of Twitter, on which they have not been trained before. The further utilization and qualitative comparison and analysis of the performance of BERT-based models specifically trained on Twitter datasets, such as TwHIN and XLM-T, will be one of the key objectives of the future work focusing on the multilingual analysis of tweets.

Though the primary focus of this paper is the analysis and the comparison of multilingual approaches as concerns the tasks of multilingual text categorization and multilingual sentiment analysis, the approach developed is applicable to any multilingual text classification task. To this end, there is a lot of additional work to perform according to the utilization and enhancement of the BERT-MC models and the ZSSC approach as they are relatively newly introduced research areas and applications and their adaptation in several domain fields and various real-world scenarios needs to be further examined. Both the proposed approaches will be further evaluated and applied in the context of a holistic environment for data-driven policy making as realized by the PolicyCLOUD project [62], where data from four different languages (Bulgarian, Italian, Spanish, and English) will be utilized and processed. In the context of this project several different real-world scenarios related with multilingual text classification tasks are addressed, such as the assessment of online propaganda and the analysis of current and future trends of radicalization actions by analyzing data gathered from social networks, websites, and blogs. Such kind of data are gathered, processed, and analyzed with the ultimate goal to implement and integrate a complete system that will transcend the barriers and obstacles provided by modern multicultural and multilingual societies especially across the European Union and provide meaningful insights and valuable information to policy makers at any level (local, regional, national, and international) to validate existing policies and revise them or even to establish new ones.

## Data Availability

Two different datasets have been used to generate the figures, tables and the supplementary figures and tables in the published article, the MARC dataset and the Twitter dataset supporting a real-world scenario. The Twitter dataset generated and analyzed during the current study is available from the corresponding author on reasonable request as it has been created under the scopes of the PoliyCLOUD project [62] and it has been uploaded in its corresponding repository. The project is not finalized yet, thus any data sharing implies its consortium approval. The MARC dataset is a public dataset available from Amazon as stated in the respective reference [56].

## Footnotes

1 https://pypi.org/project/tinysegmenter/.
